# ‘The evils are uncontrolled’: Marx’s *Capital* as a form of witnessing for child public health

**DOI:** 10.1093/pubmed/fdag037

**Published:** 2026-05-15

**Authors:** Myles Balfe

**Affiliations:** Medical Sociology, Department of Sociology, UCC, Cork, Ireland

**Keywords:** children, young people, work environment

## Introduction

Karl Marx was one of the three founders of modern Sociology, along with Max Weber and Emile Durkheim. In this short article I examine his magnum opus, *Capital*^1^ as an important, if underrecognized, work of Public Health. *Capital* is increasingly being recognized as a work of the humanities/social sciences with important lessons for the health professions^2^. It is a literary as well as a Sociological masterpiece. Its profound influence on the world stemmed at least in part from its literary power, from its horrific descriptions of workers being destroyed and exploited by capitalist entities. Marx noted in *Capital* that ‘political economy was studied…with the greatest success by medic[s]’. More particularly, I think that he felt that it was studied with the greatest success by health professionals with a strong Public Health ethos.

This article concentrates particularly on the role of children in Marx’s *Capital*. Although *Capital* does not have a dedicated chapter on children, children are focused on throughout the book. Marx focuses on children for two key reasons. Firstly, he was personally outraged by child economic exploitation. Secondly, children are also a stand-in for all workers who are vulnerable to exploitation by capitalist entities. I think that he is saying that if this system has the potential to do *this* to *this* group, it can do *this* to any group. All direct quotes in this short piece are from *Capital*. 

Marx wrote at the start of modern capitalism. During this period, advanced technology was becoming embedded within capitalist modes of production, and masses of people were employed in what Marx called ‘the factory hell’. Marx noted that child workers played an important part in the development of this system. Child workers in Marx’s time had little legal protection and were highly vulnerable to exploitation. In *Capital*, Marx documents the exploitation and ruin of children’s bodies, minds, and health, their ‘slow sacrifice’,^1^ their ‘normal decimation’, by early industrial capitalism.

*Capital* identifies the exploitation of children across a wide variety of economic sectors. Marx noted the ‘frames dwindling’ of children in the lace trade, where children began work at 6 years of age; the ‘half-starved’ children of the lucifer trade; the working to death of girls in the milliner trade; the children ‘slaughtered out-and out for the sake of their delicate finger’ in the silk trade, the destruction of girls in the rag trade.

Marx also documented the amalgamation of children with machines, arguing that children were ‘the first thing sought for by capitalists who used machinery’. Marx felt that a feature of capitalism that differentiated it from previous systems of socioeconomic production was its need to combine workers with machines- Marx calls this process ‘dissection’. He argued that capitalism seeks first to develop production systems composed of linked organic (human) and machine components. Over time, capitalism will then seek to entirely dispense with living workers and replace them with machines. This results in sectors of workers constantly losing their work as their jobs are automated away. They become a surplus reserve population for capitalism and help to keep wages and conditions down for the workers who are still in employment, while ensuring increased demand for commodities such as housing. Marx notes that if the surplus population is genuinely extra to requirements, ‘the surplus would have to die’.

Marx saw capitalism as a system that was inimical to human life and health. Consequently, *Capital* documents the serious health consequences that capitalist exploitation had for child workers. Children frequently became so tired from working that ‘neither rules of health, of cleanliness… are in the least observed’. Children in the tile-trade were forced to carry 10 tons of tiles a day. Marx notes that child workers worked ‘for a bare subsistence’ and lacked nutrients to ‘arrest starvation diseases’. They frequently had no time to eat during work. Children consequently physically degenerated and were exposed to and unable to resist a wide number of diseases, ranging from lockjaw to typhus to smallpox. Their mental health was destroyed, their work ruining them as ‘intellectual and moral beings’. Marx noted that their working conditions also ‘exhausts the nervous system to the utmost’. Whether in work or at home, child workers were frequently in highly over-crowded spaces where ‘vitiation of the air are often extreme’. Children’s ‘work for the capitalist usurped the place…of the children’s play’. *Capital* also documents that these children were sometimes raped and physically abused. They were forcibly dosed with opiates to make them docile, and, if considered to be an economic burden, sometimes killed. A medical officer noted that it was unsurprising that the average age of death of child workers in some cities was 17; another medical officer despaired of ‘my knowledge of such evils’ that those children were subjected to.

Adult workers, the parents of those children, also experienced extraordinary health damage from their working conditions. *Capital* documents that they die from pulmonary disease, kidney disease, and pneumonia. They starve to death and die of consumption. Fundamental biological processes are disrupted; Marx identifies the ‘irregularity of uterine function in stove workers’, the workers’ lives ‘uselessly tortured and shortened by the never-ending physical suffering that their mere occupation begets’. These workers have terrible living conditions, living with their children in circumstances characterised by destitution and overcrowding, exploited by landlords, and made to pay unaffordable rents. Overcrowding triggers regular disease outbreaks of amongst adults and children. 

*Capital* identifies the underlying causes of workers and children’s suffering. From the point of view of capitalists, children were ideal workers as they could be employed cheaply and were more easily controlled and deceived than adults (‘men would be less obedient’). Children were ‘cheap material for exploitation’. Marx noted that capitalism dehumanized children, like it did all workers, seeing them as ‘nothing more than personified labour-time’. By documenting the suffering of these children, Marx developed two key ideas that explained that suffering: absolute surplus value (capitalism must make children and adults work longer in order to increase profit) and relative surplus labour value (it made them work more intensively). *Capital* then argues that absolute and relative surplus are inherent features of capitalist systems that all workers are exposed to. The term surplus value is very important in *Capital*. It refers to that portion of a worker’s life force that is extracted from them by capitalist entities without recompense, even, in fact, without their knowledge.

Throughout *Capital*, Marx draws on data from contemporaneous medical and Public Health sources, from reports by doctors and surgeons who observed these children, to reports by journalists, to factory inspectors who attempted to mitigate the children’s exploitation. He quotes one surgeon, for instance, who notes, ‘I can only speak from personal observation…but I do not hesitate to assert my indignation has been aroused again and again at the sight of poor children whose health has been sacrificed to gratify the avarice of either parents or employers’. Marx recognized that health professionals had in an important role in documenting the iatrogenic health risks of capitalism; he noted in the 1844 Manuscripts that capitalism ‘does not recognize the unemployed, the starving, the wretched…these are figures for the eyes of the doctor’.^3^ Doctors identify the specific social causes of disease (one doctor notes that although his ‘official point of view is one exclusively physical, common humanity requires that the other aspect of this evil [the social causes of disease] should not be ignored’). Marx quotes the surgeon Mr. Charles Parsons, who notes that the poor health amongst workers is simply down to ‘long hours’. Doctors note that ‘the mischiefs of the trade…spring from capital…this power tells throughout the whole class’.

In effect, individuals who take a Public Health perspective to understand adult’s and children’s health become *witnessing professionals* in *Capital*.^4^ The Psychiatrist Robert Lifton notes that witnessing professionals are those who use their professional knowledge and experience to document the dangers of the catastrophe that they see in front of them. Lifton notes that witnessing professionals can ‘imagine the real’ and can become bearers of catastrophic truth- in this case, what this system was doing to adult workers and children. Lifton notes that there are two types of professional witnesses to catastrophe. The first are those that have what he calls ‘fragmentary awareness’- they can describe the horrors of the exploitation that they see in front of them and react against it. Those who have ‘formed awareness’ can go further and understand cause and effect in relation to the catastrophe. What *Capital* is doing in relation to the destruction of adult and children’s health is drawing on the accounts of health professionals who realize that there is something wrong here- that is, they have fragmentary awareness. Then, by using the accounts of those witnesses, *Capital* develops explanatory concepts such as absolute and relative surplus value- *Capital* therefore itself becomes a form of formed witness for Public Health.

Lifton also, however, recognizes another form of witness- false witness. False witnesses are those who attempt to ‘eclipse the actual catastrophe’ and by doing so can sustain or even intensify situations of malignant normality. *Capital* identifies three forms of false witness in relation to Public Health. The first are capitalists themselves, who make comments such as, in factories, ‘the boys do not suffer from the heat’, and that it is for the benefit of families that children work, as otherwise how would the family ‘pay off debts or get their furniture out of pawn’. Marx noted that when Public Health regulations increased around children’s health, capitalists drew on ‘capitalistic anthropology’ to attempt to redefine the age of childhood to suit their production needs. The second form of false witness was contained within state institutions. Whereas some state professionals, such as factory inspectors, were genuine witnesses and saw the exploitation of children as ‘the great evil’, others (who we would today consider to be subject to corporate capture) argued for the importance of being ‘reasonable’ when it came to balancing children’s health with the needs of capital. The third form of false witness in *Capital* was health professionals and Public Health professionals who were bought off by capitalist interests. Marx notes for instance that, to the despair of some factory inspectors, some ‘certifying surgeons…overstated the age of the children, agreeably to the capitalist’s greed for exploitation’. The presence of false witnesses in the system meant that efforts to regulate child Public Health in Marx’s time were often not consistent and not standard from one area or one factory to the next.

I think that in many ways it is important to think of *Capital* as not only a historical document, but an account of how capitalism will tend to operate in relation to vulnerable populations, given limited legal and Public Health constraints placed over it, and given its inherent drive towards dissection and extracting absolute and relative surplus value. Marx’s *Capital* contains lessons for Public Health professionals in the 21st century.

The first of these relates to the profession’s duty to bear witness to capitalistic suffering and exploitation. Economic inequality is exploding in the 21st century, and the world is increasingly being divided into those with wealth, workers acceptable to those with wealth, and people who are merely considered to be waste- what Marx called the lumpenproletariat. A keyword of 21st-century capitalism is deregulation, which means removing restrictions around capital and returning capitalism to a system more like that found in Marx’s time. Bearing witness to economic exploitation and its health consequences is absolutely not risk free and requires courage and ability to see clearly and to speak truth to, and against the interests of, those with economic power. Marx quotes one report where a worker notes that ‘many a man might wish to object to employing a boy, but he would perhaps become marked by it’. The worker is asked ‘marked by whom?’, to which he replied, ‘his employers’. Public Health professionals who take on economic power can experience similar risks today. Marx once said that you should never play at insurrection. Those acting as witnesses to capitalist exploitation need to have formed awareness. However, they also need to know that it was their profession that historically helped to control the health damage that was inflicted by this socioeconomic system in its early incarnation.

The second lesson I think is to understand that false witnesses who support economic exploitation and inequality, because it is in their own economic or political interests to do so, can emerge even from the ranks of health professionals. As Marx would say, no one should be naive about how human nature operates under capitalistic systems.

The third lesson is that certain groups of workers are particularly vulnerable to capitalist exploitation, and that such exploitation can have profoundly damaging effects on their health. In Marx’s time, children were one such group. Today, in advanced industrial economies, such groups include those on precarious forms of employment, those on zero-hour contracts, those on minimum wage, as well as those subjected to the erosion of labour protections and job security in the pursuit of increased profitability. In addition, many contemporary workers are subjected to forms of technological surveillance and performance monitoring that, while more sophisticated, are very similar to the highly controlled and coercive labour conditions of early industrial capitalism. Although the specific technologies and industries have changed, the underlying imperatives and mechanisms of capitalism remain broadly consistent.

Children, meanwhile, continue to experience economic exploitation in multiple ways. In some parts of the world, forms of labour exploitation persist that are comparable to those observed in Marx’s time. In more technologically advanced contexts, children are increasingly drawn into addictive systems of data extraction associated with what has been termed ‘surveillance capitalism.’^5^ In these systems, behavioural and personal data are collected and commodified for corporate profit, for the economic benefit of centimillionaires and billionaires. Such practices contribute to growing mental health pressures among young people, just as exploitative labour conditions contribute to stress, burnout, and alienation among adults. Marx would have also recognized technological capitalism’s ongoing attempts to use the data extracted from surveillance capitalism to generate self-acting machines and systems, and thereby perhaps condemn many children and adults to a future where they will forcibly decomposed into surplus populations. He noted that while capitalism often presented a positive and caring face to the world, it was completely indifferent about letting organic material go to the wall with every revolution in the means of production.

The fourth lesson is that political economy, an awareness of capitalism, is important for Public Health. Public Health researchers often do excellent work on the social determinants of health and draw attention to their problematic nature. One area where I think Marx might differ from modern Public Health professionals is that he might say that there is nothing problematic, from a system point of view, about the social determinants of health. For instance, poverty and precarious work are important social determinants that are widely recognized to undermine health. Marx would say that in a capitalist system no one should be surprised by the presence of either economic struggle or precarious work. In this system, you are meant to have billionaires at one end and people who are barely surviving on the other. This is the system working correctly and effectively, and is the natural outcome of capitalist reproduction cycles, commodification processes, and surplus value extraction procedures. If healthcare is being privatized and housing being commodified to the detriment of workers’ health, there is nothing concerning here from a system point of view; this is how the system is meant to work. I think he would say that Public Health professionals who focus on the social determinants of health without paying attention to the fact that the social determinants of health are often just symptoms of the effective functioning of capitalism do not yet have formed awareness about the nature of the system that they are in. He called this type of mid-level system awareness, awareness of social determinants of health, but lack of awareness of political economy, as *bourgeois socialism*. In Lifton’s terms, this is more than fragmentary awareness, but is not yet full understanding. Basically, he would encourage Public Health professionals not just to pay attention to the social determinants of health, but the nature of the system that often generates those social determinants.

And finally, *Capital* shows the importance of writing scientific findings with literary skill and power. It is that combination that can generate the energy needed to change some part of the world.

*Capital* is an indictment of the past, and continues to be a warning to us, the inheritors of Marx’s future.

## Notes

**Myles Balfe**, Senior Lecturer
